# Clinical characteristics, treatment outcomes, and predictors of early mortality in *Vibrio vulnificus* infection in Eastern Thailand: A 6.5-year multicenter cohort study

**DOI:** 10.1371/journal.pntd.0014353

**Published:** 2026-05-29

**Authors:** Jatapat Hemapanpairoa, Supaporn Wongduang, Tippawan Wongwian, Wichai Santimaleeworagun

**Affiliations:** 1 . Division of Pharmaceutical Care, Faculty of Pharmacy, Silpakorn University, Nakhon Pathom, Thailand; 2 Division of Clinical Pharmacy, Department of Pharmacy, Chonburi Hospital, Muang, Chonburi, Thailand; 3 Pharmacy Department, Queen Savang Vadhana Memorial Hospital, Thai Red Cross, Chonburi, Thailand; Fukuoka University Hospital: Fukuoka Daigaku Byoin, JAPAN

## Abstract

*Vibrio vulnificus* is a rapidly progressive, life-threatening pathogen endemic to coastal environments, yet clinical data from Southeast Asia remain limited despite substantial environmental contamination. We conducted a 6.5-year multicenter retrospective cohort study at two tertiary-care hospitals in Eastern Thailand to describe clinical characteristics, antimicrobial susceptibility, treatment practices, and factors associated with early mortality among adults with culture-confirmed *V. vulnificus* infection. Forty-nine patients were identified, most of whom presented with severe illness; 83.7% had bacteremia, 61.2% required mechanical ventilation, and 75.5% required vasopressor support. Skin and soft tissue infection was the most common syndrome, with necrotizing soft-tissue infection accounting for approximately two-thirds of cases. Antimicrobial susceptibility was uniformly high for fluoroquinolones, carbapenems, and third-generation cephalosporins, although susceptibility to doxycycline was unavailable. Despite high rates of empirical antimicrobial therapy (91.8%), mortality remained substantially high: 42.9% died within 48 hours, and 7-day mortality reached 49.0%. In multivariable analysis, elevated serum lactate and lack of surgical treatment were associated with early mortality, while chronic alcohol use and vasopressor requirement showed nonsignificant trends toward increased mortality. These findings highlight the fulminant nature of *V. vulnificus* infection and the narrow therapeutic window for effective intervention. In settings where necrotizing soft-tissue infection is common and patients present with advanced disease, early recognition, aggressive resuscitation, timely surgical management, and prompt initiation of antimicrobial therapy remain essential. This study provides region-specific epidemiologic and clinical insights that may inform clinical management and public health strategies in coastal communities at risk.

## Author summary

*Vibrio vulnificus* is a naturally occurring marine bacterium found in warm coastal waters and seafood such as raw oysters. Although infections are uncommon, they can progress rapidly, leading to severe soft-tissue destruction, septic shock, and death within hours. People with chronic liver disease or chronic alcohol use are at particularly high risk. Eastern Thailand is an area where *V. vulnificus* is frequently detected in seafood, yet clinical data to guide early recognition and treatment remain limited.

We reviewed six and a half years of cases from two major hospitals in Chonburi Province to better understand how this infection presents, how patients are treated, and which factors are associated with early death. Nearly half of patients died within the first week, and many died within the first 48 hours. High lactate levels at presentation were associated with more severe illness and a higher risk of death. Timely surgical removal of infected or necrotic tissue—when an infected wound was present—was associated with improved survival. Although most patients received antibiotics with in vitro activity against the bacteria, this alone was often insufficient once severe organ failure had developed.

These findings highlight the rapid and life-threatening nature of *V. vulnificus* infection, the importance of early recognition, and the need for timely access to surgical care in coastal endemic areas. The results also underscore the need for targeted public-health messaging for individuals at highest risk, particularly those with chronic liver disease or chronic alcohol use. This study provides region-specific information that may help improve clinical care and inform prevention efforts in coastal communities.

## Introduction

*Vibrio vulnificus* is a halophilic Gram-negative pathogen endemic to coastal and estuarine environments and a leading cause of rapidly progressive foodborne and wound-related infections. Human disease typically results from ingestion of contaminated seafood or exposure of open wounds to seawater, and may progress quickly to necrotizing soft-tissue infection or fulminant septicemia, particularly among individuals with chronic liver disease, diabetes, iron overload, or other immunocompromising conditions [1]. Although environmental contamination with *V. vulnificus* is well documented in tropical regions, including substantial contamination of raw oysters along the eastern Thai coastline [2], systematically collected clinical data from this region remain limited.

Optimal management requires early recognition, rapid initiation of active antimicrobial therapy, and timely surgical treatment. However, current evidence guiding clinical decisions is largely based on observational studies, antimicrobial susceptibility patterns vary by geography, and early mortality predictors remain insufficiently defined [3,4]. Existing reports from Thailand are limited to small case series and provide little insight into local treatment practices, antimicrobial susceptibility, or factors associated with early death [5,6]. To address these knowledge gaps, we conducted a multicenter retrospective cohort study of adults with culture-confirmed *V. vulnificus* infection in Chonburi Province, Eastern Thailand. This study aimed to describe the clinical characteristics, antimicrobial susceptibility profiles, and treatment patterns of patients with culture-confirmed *V. vulnificus* infection in Eastern Thailand, and to explore clinical factors associated with early mortality in this cohort.

## Materials and methods

### Ethics statement

The study protocol was approved by the Institutional Review Board of the Chonburi Hospital Clinical Research Center (CBH-IRB No. 56/68/O/h3) and the Ethics Committee of Queen Savang Vadhana Memorial Hospital (IRB No. 032/2568). The requirement for informed consent was waived due to the retrospective nature of the study and minimal risk to participants. All data were anonymized prior to analysis. The study was conducted in accordance with the principles of the Declaration of Helsinki.

### Study design and setting

We conducted a retrospective two-center cohort study with systematic chart review at two tertiary-care hospitals in Chonburi Province, Thailand. All eligible cases diagnosed between January 1, 2019, and June 30, 2025 were included.

### Participants

We included adults aged ≥18 years with culture-confirmed *V. vulnificus* infection from any clinical specimen (blood, wound, or other normally sterile sites). Cases were identified through microbiology laboratory databases and verified through electronic medical records. Only the first episode per patient was included.

### Data collection and variable definitions

We extracted demographic characteristics, comorbidities, exposure history, infection site, clinical severity indicators, antimicrobial susceptibility results, and antimicrobial therapy. Severity indicators—platelet count, serum lactate, total bilirubin, serum creatinine, vasopressor use, and mechanical ventilation—were recorded on the index date (defined as the date of positive culture collection).

The site of infection was determined based on physician documentation. Clinical syndromes were classified as skin and soft tissue infection, primary septicemia, or gastroenteritis. These syndromes represent heterogeneous clinical presentations with distinct pathophysiology and management.

Chronic liver disease was defined as documented cirrhosis, chronic hepatitis (including hepatitis B or C infection), or other chronic hepatic conditions recorded in the medical record. Hepatitis B virus infection, hepatitis C virus infection, and chronic alcohol use were recorded as separate variables and may overlap with chronic liver disease.

Exposure history was extracted from physician documentation in the medical record and included reported trauma, wound exposure, seafood consumption, or seafood handling when available.

Empirical antimicrobial therapy was defined as antimicrobial agents administered prior to the availability of microbiological identification and susceptibility results. Antimicrobial therapy after microbiological confirmation was defined as therapy administered after pathogen identification and susceptibility results became available. Given the heterogeneity of treatment regimens and limitations in susceptibility data, antimicrobial therapy was analyzed descriptively.

Surgical treatment was defined as operative interventions, including debridement, fasciotomy, or amputation.

### Outcomes

The primary outcome was 7-day all-cause mortality. Secondary outcomes included 48-hour, 14-day, and 30-day all-cause mortality, as well as in-hospital mortality, defined as death from any cause during the index hospitalization.

### Statistical analysis

Continuous variables were summarized as median (interquartile range, IQR), and categorical variables as number (percentage). Group comparisons used the Mann–Whitney U test for continuous variables and the Chi-square or Fisher’s exact test for categorical variables, as appropriate. Variables associated with 7-day mortality at *p* < 0.10 in univariate analysis, or considered clinically relevant, were included in a multivariable logistic regression model. Statistical significance was defined as *p* < 0.05. Analyses were performed using IBM SPSS Statistics version 27.0 (IBM Corp., Armonk, NY, USA).

Given the number of outcome events, a parsimonious model was used to reduce the risk of overfitting. Analyses of factors associated with early mortality were considered exploratory and hypothesis-generating.

## Results

### Clinical characteristics

During the 6.5-year study period, 49 patients with culture-confirmed *V. vulnificus* infection were identified. Of these, 38 patients (77.6%) had positive blood cultures only, 8 (16.3%) had positive wound cultures only, and 3 (6.1%) had both blood and wound isolates. The median age was 55 years (IQR 46.5–65.5). Chronic liver disease was the most common comorbidity (20/49, 40.8%). Chronic alcohol use was documented in 19 patients (38.8%). However, the underlying etiologies of liver disease were heterogeneous and not consistently documented in the medical records. Severe clinical presentation was frequent: 30 patients (61.2%) required mechanical ventilation and 37 (75.5%) required vasopressor support.

Skin and soft tissue infection (SSTI) was the most common syndrome (31/49, 63.3%). Among SSTI cases, necrotizing fasciitis occurred in 21 patients (67.7%), cellulitis in 5 (16.1%), and unspecified SSTI in 5 (16.1%) (Table 1).

**Table 1:** Baseline characteristics of patients with *Vibrio vulnificus* infection (N = 49).

Characteristics	Number (%)
**Age (years)**	Median 55 (IQR 46.5–65.5)
**Male**	28 (57.1)
**Comorbidities**	
Chronic liver disease	20 (40.8)
HCV infection	7 (14.3)
HBV infection	2 (4.1)
HIV infection	1 (2.0)
Hypertension	11 (22.4)
Diabetes	10 (20.4)
Gastrointestinal disorder	5 (10.2)
Chronic kidney disease	4 (8.2)
Ischemic heart disease	3 (6.1)
Hematologic disorders	3 (6.1)
Dyslipidemia	2 (4.1)
Chronic heart failure	1 (2.0)
Papillary renal cell carcinoma	1 (2.0)
Hepatocellular carcinoma	1 (2.0)
**Chronic alcohol use**	19 (38.8)
**Exposure history**	
Trauma without wound	3 (6.1)
Wound exposure	2 (4.1)
Seafood consumption	2 (4.1)
Seafood handling	1 (2.0)
**Severity indicators**	
Platelet (×10³/µL)	Median 72 (IQR 42–137.5)
Lactate (mmol/L)	Median 9.8 (IQR 6.3–16.1)
Bilirubin (mg/dL)	Median 2.1 (IQR 1.0–4.3)
Serum creatinine (mg/dL)	Median 2.0 (IQR 1.3–2.7)
Mechanical ventilation	30 (61.2)
Vasopressor requirement	37 (75.5)
**Bacteremia**	41 (83.7)
**Site of infection**	
Skin/soft tissue infection	31 (63.3)
Primary bacteremia	15 (30.6)
Gastroenteritis	3 (6.1)

**Fig 1:**
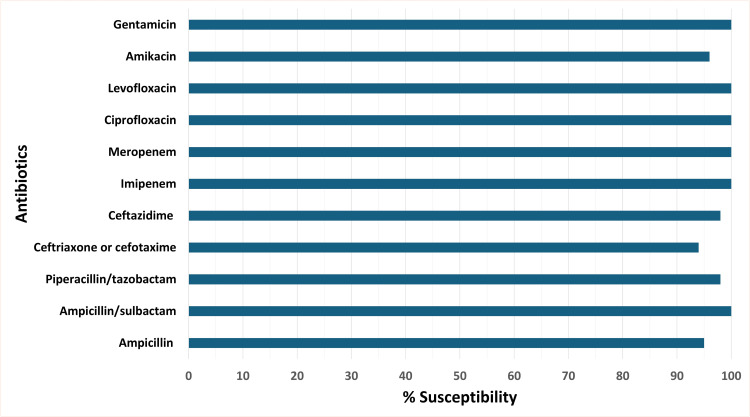
Antimicrobial susceptibility of *Vibrio vulnificus* isolates (N = 49). Note: Susceptibility testing was not performed for all isolates for certain antimicrobial agents. The number of isolates tested for each drug was: ampicillin (N = 38), ampicillin–sulbactam (N = 37), and piperacillin–tazobactam (N = 44). Susceptibility testing for doxycycline was not available.

All isolates tested were susceptible to gentamicin, ciprofloxacin, levofloxacin, meropenem, and imipenem. Susceptibility to third-generation cephalosporins was high (94% for cefotaxime/ceftriaxone; 98% for ceftazidime). Susceptibility testing for doxycycline was not available (Fig 1).

### Treatments

Empirical antimicrobial therapy was administered to 48 of 49 patients (98.0%); one patient did not receive antimicrobial therapy prior to death. Regimens most commonly included third-generation cephalosporins.

Twenty-five patients (51.0%) received antimicrobial therapy after microbiological confirmation, including 11 who received monotherapy and 14 who received combination therapy. The remaining patients died before microbiological confirmation became available. Six patients were transitioned to oral agents before discharge.

Surgical treatment was performed in 23 patients. The majority underwent surgical debridement (16/23, 69.6%), followed by debridement with fasciotomy (4/23, 17.4%) and amputation (3/23, 13.0%). Detailed treatment characteristics are provided in Table 2.

**Table 2:** Treatment characteristics of patients with *Vibrio vulnificus* infection (N = 49).

Treatment characteristics	Number (%)
**Empirical antimicrobial therapy**	48 (98.0)
**Single-regimen empirical therapy (N = 34)**	
Third-generation cephalosporins + clindamycin	13 (38.2)
Third-generation cephalosporins alone	6 (17.6)
Meropenem	5 (14.7)
Third-generation cephalosporins + metronidazole	3 (8.8)
Amoxicillin/clavulanic acid	2 (5.9)
Meropenem + clindamycin	1 (2.9)
Third-generation cephalosporins + doxycycline	1 (2.9)
Third generation cephalosporins + clindamycin + doxycycline	1 (2.9)
Third generation cephalosporins + metronidazole + doxycycline	1 (2.9)
Third generation cephalosporins + clindamycin + cefazolin	1 (2.9)
**Two-agent empirical therapy (N = 13)**	
Third generation cephalosporins + amikacin (single dose)	9 (69.2)
Third generation cephalosporins+ amikacin + clindamycin	2 (15.4)
Third generation cephalosporins + clindamycin + levofloxacin	1 (7.7)
Third generation cephalosporins + ciprofloxacin	1 (7.7)
**Three-agent empirical therapy (N = 1)**	
Third generation cephalosporins + amikacin + levofloxacin	1 (100)
**Antimicrobial therapy after microbiological confirmation (N = 25)**	
**Monotherapy (N = 11)**	
Third generation cephalosporins	6 (54.5)
Levofloxacin	3 (27.3)
Meropenem	1 (9.1)
Ertapenem	1 (9.1)
**Combination therapy (N = 14)**	
Third generation cephalosporins + doxycycline	5 (35.7)
Third generation cephalosporins + fluoroquinolones	5 (35.7)
Meropenem + doxycycline	1 (7.1)
Meropenem + fluoroquinolones	1 (7.1)
Fluoroquinolones + doxycycline	1 (7.1)
Third generation cephalosporins + fluoroquinolones + doxycycline	1 (7.1)
**Surgical treatment (N = 23)**	
Debridement	16 (69.6)
Debridement + fasciotomy	4 (17.4)
Amputation	3 (13.0)
Duration of antibiotics (days)	Median 7 (IQR 2–16)

**Note:** Third-generation cephalosporins in this study included ceftriaxone, cefotaxime, and ceftazidime. Fluoroquinolones included ciprofloxacin and levofloxacin. “Single dose” refers to a one-time administration of amikacin at presentation. One patient did not receive empirical antimicrobial therapy prior to death.

### Outcomes and factors associated with mortality

Seven-day all-cause mortality was 49.0% (24/49). Early mortality within 48 hours occurred in 21 patients (42.9%). Both 30-day and in-hospital mortality were 63.3% (31/49).

In univariate analysis, male sex, hypertension, chronic alcohol use, elevated lactate, mechanical ventilation, vasopressor requirement, and absence of surgical treatment were associated with 7-day mortality. These variables were included in multivariable analysis based on statistical or clinical relevance.

In the multivariable logistic regression model, surgical treatment was associated with reduced 7-day mortality (adjusted OR 0.19; 95% CI 0.04–0.95; *p* = 0.044), while higher lactate levels were associated with increased mortality (adjusted OR 1.15 per mmol/L; 95% CI 1.01–1.31; *p* = 0.036). Chronic alcohol use and vasopressor requirement showed non–significant trends toward higher mortality (Table 3).

**Table 3:** Factors associated with 7-day all-cause mortality among patients with *Vibrio vulnificus* infection (N = 49).

Characteristics	Death (n = 24)	Survival (n = 25)	Unadjusted OR (95% CI)	*P* value	Adjusted OR (95% CI)	*P* value
Male sex	17 (70.8%)	11 (44.0%)	3.09 (0.95–10.08)	0.085	–	–
Hypertension	2 (8.3%)	9 (36.0%)	0.16 (0.03–0.85)	0.037	–	–
Chronic alcohol use	13 (54.2%)	6 (24.0%)	3.74 (1.11–12.67)	0.042	4.82 (0.89–26.21)	0.069
Lactate (mmol/L)	Median 15.3 (IQR 8.8–20)	Median 7.1 (IQR 4.6–11.5)	–	<0.001	1.15 (1.01–1.31)	0.036
Mechanical ventilation	18 (75.0%)	12 (48.0%)	3.90 (1.10–13.80)	0.040	–	–
Vasopressor requirement	21 (87.5%)	16 (64.0%)	5.91 (1.12–31.20)	0.039	8.36 (0.72–97.56)	0.090
Surgical treatment	7 (29.2%)	16 (64.0%)	0.23 (0.07–0.77)	0.022	0.19 (0.04–0.95)	0.044

Note. Candidate variables were selected based on clinical relevance and univariate analysis. Male sex and hypertension were evaluated in multivariable analysis but were not independently associated with mortality and did not improve model fit; therefore, they were not retained in the final model. Mechanical ventilation was excluded due to collinearity with vasopressor use. Given the limited number of outcome events, a parsimonious model was used to reduce the risk of overfitting. The multivariable model may be subject to instability due to the limited number of outcome events, and results should be interpreted as exploratory. The final multivariable model included chronic alcohol use, lactate level, vasopressor requirement, and surgical treatment.

## Discussion

This study provides new clinical evidence on *V. vulnificus* infection from Eastern Thailand, where systematic clinical data remain limited despite well-documented environmental contamination [1–3]. We observed exceptionally high and rapidly occurring mortality, with nearly half of all deaths occurring within the first 48 hours. These findings are consistent with reports from multiple geographic regions [7,8], including data from Thailand in which 58.6% of patients with *V. vulnificus* septicemia died within 24 hours of presentation [5]. Collectively, these observations underscore the fulminant nature of *V. vulnificus* infection and the extremely narrow window for effective intervention.

Most patients in our cohort presented with severe disease, aligning with previous descriptions of rapid progression and early septic shock in *V. vulnificus* infection [5,6,8]. The predominant clinical syndromes—necrotizing soft-tissue infection, primary septicemia, and gastroenteritis—were consistent with established manifestations, and no atypical presentations were identified [1].

Elevated lactate levels and lack of surgical treatment were associated with mortality in this cohort. Lactate is a recognized marker of global hypoperfusion and metabolic stress, and its strong association with mortality has been documented across diverse septic populations [9]. In *V. vulnificus* infection, markedly elevated lactate likely reflects rapid circulatory collapse combined with the burden of underlying comorbidities [1,10]. Surgical treatment was associated with improved survival, consistent with international guidelines for necrotizing soft-tissue infections emphasizing early surgical intervention [11,12]. Prior retrospective studies likewise identified delayed surgery as a major predictor of mortality in *V. vulnificus* sepsis and soft-tissue infections [10,13]. However, surgical treatment was feasible primarily in patients with focal SSTI, particularly necrotizing fasciitis, and not in those with primary bacteremia. This difference in clinical applicability likely contributed to poorer outcomes among patients without an operable source and underscores that surgical treatment should not be interpreted as a uniform prognostic factor across all clinical syndromes. In the multivariable analysis, the wide confidence interval for vasopressor requirement indicates substantial imprecision, likely due to the limited number of outcome events, and the findings should be interpreted with caution.

Chronic alcohol use, vasopressor requirement, and mechanical ventilation were significantly associated with mortality in univariate analysis, although these did not remain independent predictors after adjustment—likely due to overlap with overall illness severity. Nonetheless, these associations are biologically and clinically plausible, given the well-established impact of alcohol-related immune dysfunction, hepatic impairment, and hemodynamic instability in severe *V. vulnificus* infection [10,14].

Most patients received empirical therapy; however, consistent with previous evidence, the choice of active antimicrobial agents did not clearly influence survival. Meta-analytic data suggest that antimicrobial regimen selection has limited effect on mortality once severe disease is established [10]. Among patients who survived long enough to receive antimicrobial therapy after microbiological confirmation, outcomes did not differ significantly between monotherapy and combination regimens [10,15]. A retrospective study from Taiwan reported lower mortality with combination therapy—either a third-generation cephalosporin plus minocycline or a fluoroquinolone—compared with cephalosporin monotherapy (14% vs. 61%) [16], suggesting potential benefit in selected patients, but these findings remain non-definitive.

Antimicrobial susceptibility patterns in our cohort were favorable and consistent with previous reports showing uniformly high susceptibility to β-lactams, fluoroquinolones, and tetracyclines [7,17]. A major limitation was the lack of doxycycline susceptibility testing, as recommended in CLSI M45, which is relevant given that doxycycline is a component of guideline-recommended therapy for *V. vulnificus* infection [11,18–20]. Although susceptibility patterns were reassuring, the rapid deterioration observed in many patients highlights the need for further investigation into optimal antimicrobial strategies in this setting.

The epidemiologic characteristics of this cohort, particularly the high prevalence of chronic liver disease and chronic alcohol use, are consistent with established global risk factors for severe *V. vulnificus* infection [1,7]. Exposure histories were heterogeneous and incompletely documented, with only a minority of patients having recorded exposure information. This likely reflects limitations inherent to retrospective data collection and may have led to under-ascertainment of clinically relevant exposures. Therefore, exposure-related findings should be interpreted with caution. Previous studies similarly report that many patients lack a clearly identifiable exposure source [7,21,22].

This study has several limitations. Many patients experienced very early mortality, which may have introduced survival bias and limited the ability to evaluate the impact of antimicrobial therapy or surgical treatment. Although lactate levels were measured at initial presentation and patients with necrotizing fasciitis underwent surgical treatment on the first day of admission, the timing of other clinical assessments and interventions was not fully standardized, contributing to variability in severity evaluation. Severity scores such as Sequential Organ Failure Assessment and Acute Physiology and Chronic Health Evaluation II were not consistently available, limiting adjustment for baseline illness severity in the multivariable model. Classification of clinical syndromes relied on physician documentation and may be subject to misclassification. In addition to the fulminant nature of the disease, delayed presentation or delayed access to medical care may also have influenced clinical outcomes in some patients. However, these factors were not systematically collected and therefore could not be formally evaluated in this study. In addition, the cohort included a high proportion of patients with chronic liver disease and chronic alcohol use—reflecting the risk characteristics of individuals who develop *V. vulnificus* infection rather than the general population—which may limit generalizability to populations with different underlying risk profiles. The specific etiologies of chronic liver disease were not consistently documented, and the contribution of different causes, including alcohol-related liver disease and viral hepatitis, could not be reliably determined due to potential overlap. Child–Pugh classification was also not consistently available in the medical records, limiting assessment of the relationship between liver disease severity and clinical outcomes. Exposure history was incompletely documented, with only a minority of patients having recorded exposure information, which may have led to under-ascertainment of clinically relevant exposures. Variability in MIC testing, particularly for tetracyclines, further constrained interpretation of antimicrobial susceptibility patterns. The multivariable model may be subject to instability and overfitting due to the limited number of outcome events relative to the number of predictors. The heterogeneous clinical syndromes and limited adjustment for baseline severity may further affect the robustness of the findings. Therefore, the results should be interpreted as exploratory and hypothesis-generating. Despite these limitations, the study provides important insights into a rapidly fatal infection in a setting where systematically collected clinical data remain scarce.

## Conclusion

In this retrospective cohort from Eastern Thailand, *V. vulnificus* infection was associated with extraordinarily high early mortality, with nearly half of deaths occurring within the first 48 hours of hospital admission. Elevated lactate at presentation and lack of surgical treatment were associated with 7-day mortality; however, these findings are exploratory and should be interpreted in the context of study limitations. Despite generally favorable antimicrobial susceptibility, the rapid clinical deterioration observed in many patients highlights the importance of early recognition and timely clinical management.

This study underscores the need for heightened clinical awareness, early diagnosis, and prompt surgical treatment in patients with surgically amenable infections, as well as targeted public health interventions for at-risk populations, particularly individuals with chronic liver disease or chronic alcohol use in coastal endemic regions. Prospective studies and enhanced surveillance are warranted to further clarify risk factors and improve clinical outcomes in *V. vulnificus* infection.
